# Organic Thermoelectric Nanocomposites Assembled via Spraying Layer-by-Layer Method

**DOI:** 10.3390/nano13050866

**Published:** 2023-02-25

**Authors:** Seojin Kim, You Young Byun, InYoung Lee, Woohyeon Cho, Gyungho Kim, Mario Culebras, Junho Jang, Chungyeon Cho

**Affiliations:** 1 Department of Carbon Convergence Engineering, College of Engineering, Wonkwang University, Iksan 54538, Republic of Korea; 2 Core Facility for Supporting Analysis & Imaging of Biomedical Materials, Wonkwang University, Iksan 54538, Republic of Korea; 3 DMT Company, 60, Wanggungnonggong Danji-Gil, Wanggung-Myeon, Iksan 54576, Republic of Korea; 4 Institute of Materials Science (ICMUV), University of Valencia, 46980 Paterna, Spain; 5 Wearable Platform Materials Technology Center (WMC), Department of Materials Science and Engineering, Korea Advanced Institute of Science and Technology (KAIST), Daejeon 34141, Republic of Korea

**Keywords:** layer-by-layer, spraying, thermoelectric, carbon nanotubes, polymer nanocomposites

## Abstract

Thermoelectric (TE) materials have been considered as a promising energy harvesting technology for sustainably providing power to electronic devices. In particular, organic-based TE materials that consist of conducting polymers and carbon nanofillers make a large variety of applications. In this work, we develop organic TE nanocomposites via successive spraying of intrinsically conductive polymers such as polyaniline (PANi) and poly(3,4-ethylenedioxy- thiophene):poly(styrenesulfonate) (PEDOT:PSS) and carbon nanofillers, and single-walled carbon nanotubes (SWNT). It is found that the growth rate of the layer-by-layer (LbL) thin films, which comprise a PANi/SWNT-PEDOT:PSS repeating sequence, made by the spraying method is greater than that of the same ones assembled by traditional dip coating. The surface structure of multilayer thin films constructed by the spraying approach show excellent coverage of highly networked individual and bundled SWNT, which is similarly to what is observed when carbon nanotubes-based LbL assemblies are formed by classic dipping. The multilayer thin films via the spray-assisted LbL process exhibit significantly improved TE performances. A 20-bilayer PANi/SWNT-PEDOT:PSS thin film (~90 nm thick) yields an electrical conductivity of 14.3 S/cm and Seebeck coefficient of 76 μV/K. These two values translate to a power factor of 8.2 μW/m·K^2^, which is 9 times as large as the same films fabricated by a classic immersion process. We believe that this LbL spraying method will open up many opportunities in developing multifunctional thin films for large-scaled industrial use due to rapid processing and the ease with which it is applied.

## 1. Introduction

As the use of electronic devices increases with the advancement of science, technology, and industry in modern society, the demand for energy has also rapidly increased [1,2]. Environmental issues regarding traditional energy sources such as fossil fuels have led to the stimulation of many researchers to find alternative candidates [3,4,5]. To date, various energy harvesting techniques using mechanics, electronics, magnetics, heat, and biochemistry have been developed [6,7,8]. Among these, thermoelectrics (TEs), which directly convert the electricity from thermal energy induced by cooling or heating or other waste heat, have been recognized as a viable and renewable energy harvesting technology [9,10,11]. The performance of TE devices can be evaluated by the dimensionless figure of merit (ZT), as per the following equation:

ZT = S^2^·σ·T/k (1)

where S, σ, k, and T are Seebeck coefficient (also called the thermopower), electrical conductivity, thermal conductivity, and absolute temperature, respectively. The power factor (PF), defined as S*^2^·*σ, is also used to evaluate the TE efficiency. According to this Equation (1), the TE materials with high PF and low k make the high-performance TE devices with large ZT value [12,13,14].

The inorganic-based materials (for example, Bi_2_Te_3_, PbTe, Sb_2_Te_3_, etc.) have been widely used because of their high TE performance (i.e., high ZT) [12,15,16,17]. However, these materials have several drawbacks such as toxicity, high cost, poor mechanical flexibility, scarcity of raw materials, and difficulty in processing, which hinder widespread application [12,18]. Meanwhile, organic-based TE materials that contain intrinsically conductive polymers or their composites incorporated with carbon nanofillers, such as graphene and/or carbon nanotubes (CNTs), have been proved to be promising alternatives for inorganics due to their multiple advantages including low cost, solution-based processability, light weight, and flexibility [19,20,21,22,23,24]. Although the organic TE materials typically exhibit low k in the range of 0.1~5 W/m·K, which is ideal for high-performance TE devices, the obtained ZT is far below relative to that of inorganic-based composites, due to their low S and σ values. To overcome these, the strategies of fabricating composites, where carbon materials are compounded in conducting polymers matrix, have been suggested, which have exhibited reasonably good TE behaviors [1,10,12,25].

Several approaches for fabrication of organic TE composites have been made using simple mixing, solvent thermal method, template-directed in situ polymerization, polymer emulsion, and electrodeposition [26,27,28,29,30]. However, these methods have shown the limits in controlling the nanoscale film structure/property, which ultimately produces low PF [31,32]. Our group has demonstrated that the synergistic combination of polyelectrolytes and carbon nanofillers via layer-by-layer (LbL) assembly creates highly ordered nanocomposites with large PF [3,33]. The LbL method has been utilized for developing multifunctional thin films including bio-film inhabitation, flame retardant, gas sensors, and solar cells [18,34]. LbL coatings are mainly based on electrostatic interactions through charge compensation between oppositely charged components, but other interactions including hydrophobic, van der Waals interactions, π–π interactions, and hydrogen bonding are also employed to form ultra-thin films on various types of substrates [35,36,37,38,39].

In this regards, the LbL assembly method provides a facile technology to obtain highly ordered nanocomposites with precisely controlled thin-film architectures at a molecular level [40,41,42]. The dip coating method is the one of the most widely used LbL techniques due to its simple processing, but limited sample sizes and time-consuming steps to achieve the desired properties have remained to be solved to expand its wider applications into commercial areas [31]. LbL spraying technique has been recognized as a promising alternative to the traditional dip coating method. Spray-assisted deposition process offers faster coating, typically taking a few seconds, while the deposition time for dipping is 5 to 20 min to complete each layer. In addition, the resultant multilayer thin films assembled via the spray-LbL technique preserve the chemical nature of each component with no loss of structural control [43]. Therefore, spray coating offers an opportunity to circumvent inherent drawbacks in dip-assisted assembly.

Herein, the hybrid nanocomposites were fabricated with polyaniline (PANi) and poly(3,4-ethylenedioxythiophene) polystyrene sulfonate (PEDOT:PSS)-stabilized single-walled carbon nanotube (SWNT) using the LbL assembly technique. The multilayer growth behavior, films’ morphology, and thermoelectric performance via successive spraying were compared to classic dipping. The spray-based LbL assembly produced a higher electrical conductivity with a similar Seebeck coefficient, translating to a larger power factor relative to a classic immersion process. A 20-bilayer PANi/SWNT-PEDOT:PSS (~90 nm in thickness) exhibited a power factor of 8.2 μW/m·K^2^, which is 9 times as large as the same films fabricated by the dipping process.

## 2. Materials and Methods

### 2.1. Materials

Polyaniline (PANi, MW = 50,000 g/mol) and N,N-dimethyl acetamide (DMAC) were purchased from Sigma-Aldrich (Milwaukee, WI, USA). PANi solution for multilayer deposition was prepared as follows: 0.1 g of PANi powder was first dissolved in 30 g of DMAC under continuous stirring for 30 min and then sonicated for 90 min, after which the mixture was stirred for overnight. Then, 270 mL of pH 3.0 water was added to the PANi–DMAC solution, and the PANi bath was adjusted to pH 2.5 prior to LbL coating. Poly(3,4-ethylenedioxy thiophene):polystyrene sulfonate (PEDOT:PSS, Clevios P) was obtained from Heraeus Precious Metals (Hanau, Germany). Single-walled carbon nanotubes (SWNT) were provided from Cheap Tubes Inc. (Brattleboro, VT, USA). All chemicals were used as received without purification. Deionized (D.I) water with a specific resistance greater than 18 MΩ was used in all aqueous solutions. In order to make stable SWNT-PEDOT:PSS suspensions in water, 0.2 g of SWNT was sonicated in 2 wt% of PEDOT:PSS solution using bath sonicators for 1 h, and then tip sonication (Bandelin Sonopuls) was followed for 30 min with 50 W power in an ice water bath.

### 2.2. Layer-by-Layer Assembly

In order to fabricate the multilayer thin films using traditional dip coating LbL process, substrates such as glass slides or poly(ethylene terephthalate) (PET) were first immersed into the positively charged PANi solution for 5 min and rinsed with D.I water. The positively charged sample was then dipped into the SWNT-PEDOT:PSS suspension for 5 min, along with a rinsing step, which results in one deposition sequence of a PANi/SWNT-PEDOT:PSS bilayer (BL). After this initial BL was deposited, the remaining number of BLs were created using 1 min deposition until the desired number of BLs was achieved. For a spray-based coating, the substrates were vertically oriented, with a distance of 15 cm from the substrate. Each component was sprayed for 15 sec with no rinse steps between each spray coating process. The thin films made by both spraying and dipping process were air-dried overnight, prior to analyses.

### 2.3. Thin Film Characterization

The thickness of the thin films deposited on silicon wafers was measured by a NanoMap-PS contact mode stylus Profilometer. Top surface images of the nanocomposites were analyzed by atomic force microscopy (AFM) (Nanostation IITM Surface Imaging Systems, Herzogenrath, Germany) in the non-contact mode at a scan rate of 1 Hz and S-4800 field emission scanning electron microscope (Core Facility for Supporting Analysis & Imaging of Biomedical Materials, FE-SEM, Hitachi, Japan). The UV–vis absorbance spectra of the thin films deposited on quartz slides were recorded using a Shimadzu UV-1900 spectrophotometer (Japan).

### 2.4. Thermoelectric Property Measurements

The sheet resistance of thin films deposited on glass slides was acquired using a four-point probe system (CMT-100S, Advanced Instrument Technology). The probe tips were 0.4 mm in diameter with a 0.72 mm tip spacing between the probes. The electrical conductivity was calculated by taking the inverse of the product of the sheet resistance and the thickness of the thin films. Both carrier concentration and mobility were analyzed using a Hall effect measurement system (Ecopia, HMS-3000, Gyeonggi-do, South Korea). A magnetic field of ±1 T was applied to 2 × 2 cm square samples in the van der Pauw geometry. Carrier mobility was calculated with the Drude-Sommerfeld free electron model, σ = neμ, where σ, n, e, and μ are the electrical conductivity, charge carrier concentration, electronic charge, and carrier mobility, respectively [44]. The Seebeck coefficient of the thin films deposited on glass slides was determined with a custom-built four-point probe setup, where electrical voltage and temperature difference are measured by two copper wires and two T-type thermocouples. The reported Seebeck coefficient values were obtained from the slope of the linear fitting of ∆T and ∆V.

## 3. Results

**Figure 1:**
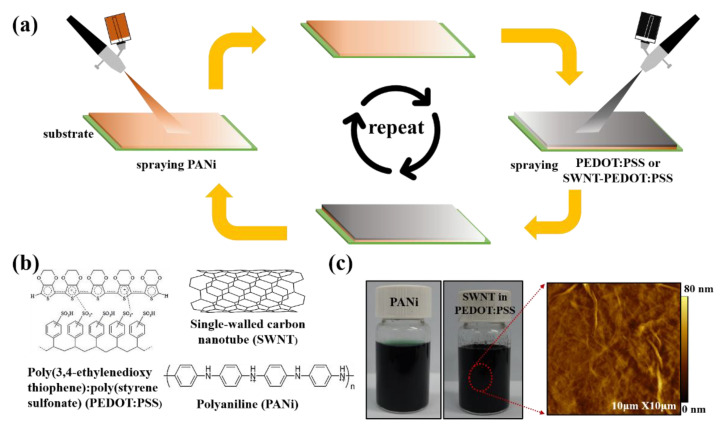
(**a**) Illustration for fabrication process of LbL-thermoelectric nanocomposites via spray approach, (**b**) chemical structures of PEDOT:PSS, SWNT, and PANi used in this study, and (**c**) photograph of PANi and SWNT, stabilized in PEDOT:PSS, along with a corresponding AFM image that shows uniformly dispersed SWNT in PEDOT:PSS solution.

Figure 1a is a schematic illustration of the LbL deposition process to form the multilayer nanocomposite (i.e., PANi/SWNT-PEDOT:PSS) thin film via the spray coating method. Detailed conditions are explained in the Materials and Methods section. Figure 1b represents the chemical structures of conducting polymers (i.e., PANi and PEDOT:PSS) and SWNT used in this work. The strong electrostatic interaction between positively charged PANi and negatively charged PEDOT:PSS is the main driving force for creating the multilayer thin films. SWNTs are assembled through π–π interactions and Van der Walls forces, with the polymers carried along [3]. Figure 1c shows photo images of dark-green PANi and SWNT, stabilized in PEDOT:PSS solutions. Sonicating carbon nanotubes in an anionic polymer creates a homogeneously dispersed suspension, as confirmed by an atomic force microscope (AFM) image in which nanotubes are uniformly distributed on the surface. By increasing the spraying time per each solution from 5 to 30 s, it turned out that the optimum spraying time for spray-based multilayer system was found to be 15 s per solution, which was enough time to uniformly coat on the substrates.

### 3.1. Growth Behavior

**Figure 2:**
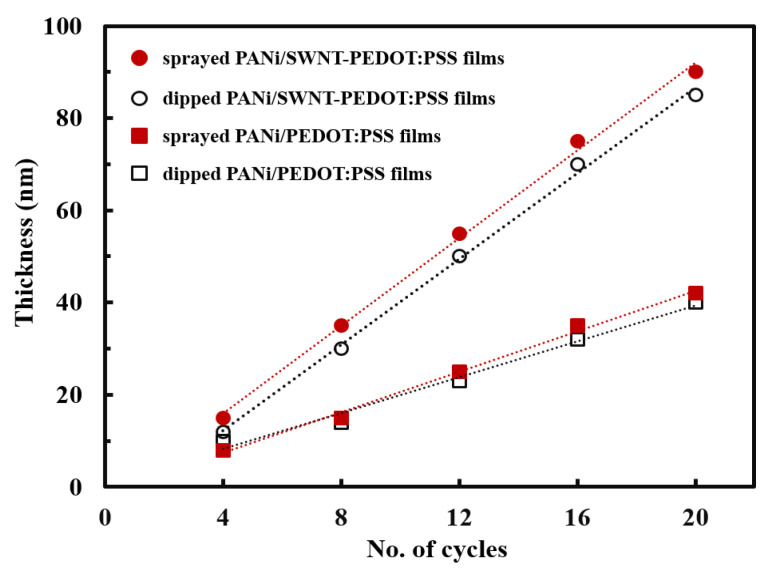
Thickness of the films with and without SWNT deposited via dipping and spraying method. The symbols represent the following: (circles) PANi/SWNT-PEDOT:PSS assemblies built by the spraying process (closed symbols) and traditional dipping approach (open symbols); (squares) PANi/PEDOT:PSS films formed by the spraying process (closed symbols) and traditional dipping method (open symbols).

In an effort to investigate how the growth behavior is influenced by coating methods, the thickness of the thin films deposited on glass substrates was measured with a profilometer after every four cycles (i.e., four BLs) (Figure 2). All multilayer thin films with and without carbon nanotubes deposited grew linearly proportional to the number of deposition cycles. The thin films made with SWNT were grown thicker than PANi/PEDOT:PSS films, which is most likely due to the three-dimensional structure of nanotubes. Interestingly, the film thickness of the spray-coated samples exhibited the slightly larger growth rate (4.5 nm per BL) relative to that of dip-coated ones (4.0 nm per BL). Deposition times for achieving 20 BLs were 90 min for the immersion process, while it only took 10 min in the case of the spraying method. This emphasizes that the spray coating speeds up the whole build-up process with the multilayer structure unaffected. The difference in growth behavior between two deposition approaches is likely due to a rinse step. As explained in the Materials and Methods section, the dip coating procedure had intermediate rinse steps between each deposition, which remove excessively and physically absorbed components. This results in a relatively thinner PANi/SWNT-PEDOT:PSS structure, as compared to that made by spraying method in the absence of rinsing. In conventional LbL assembly, the deposition steps require the rinsing process to remove loosely bound polyelectrolyte chains on the film surface and avoid any risk of cross-contamination of the assembled multilayers due to the formation of aggregates by interacting with the oppositely charged components to be deposited. However, as reported by others, the rinsing steps could be skipped in spray-LbL assembly because the shear forces on the film surface and the droplets draining off the surface remove the loosely bound materials [45]. In the present study, the LbL films with no rinse steps between each deposition exhibit high thermoelectric properties without sacrificing a highly conjugated carbon network that is commonly associated with a traditional LbL deposition, as discussed below.

**Figure 3:**
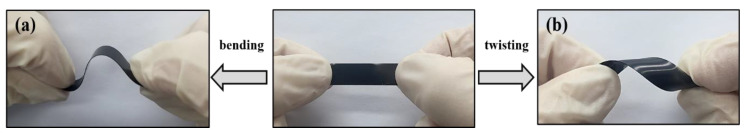
Photo images of 20 BL PANi/SWNT-PEDOT:PSS thin films deposited on the PET substrates by spraying coating method, demonstrating its flexibility by (**a**) bending and (**b**) twisting.

Figure 3 shows photographs of PANi/SWNT-PEDOT:PSS thin films with 20 BLs spray-coated on PET substrates during various mechanical deformation such as bending (Figure 3a) and twisting (Figure 3b), which highlights the high mechanical flexibility, indicating stability of multilayer thin film without any delamination. Although an investigation on the effect of electrical properties upon external stimuli is not focused on the present study, a 20 BL PANi/SWNT-PEDOT:PSS nanocomposite exhibited that the resistance increased only 10% after 100 cycles of bending (with 1.5 cm radius) and twisting.

### 3.2. Thermoelectric Performances

**Figure 4:**
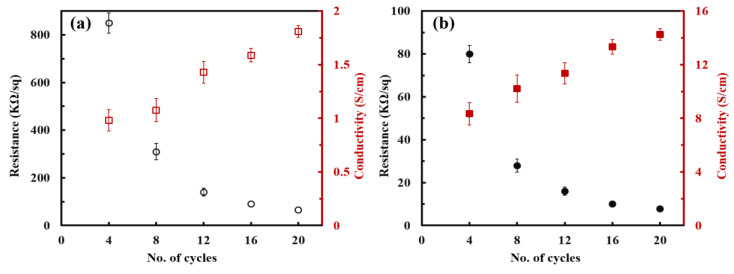
Sheet resistance and electrical conductivity of PANi/SWNT-PEDOT:PSS thin films that are coated by (**a**) traditional dipping and (**b**) spraying method.

The electrical properties of PANi/SWNT-PEDOT:PSS thin films assembled by classic LbL and spray-LbL methods were analyzed as a function of BL deposited to investigate as to how coating technologies affect the thermoelectric performances. The sheet resistance of dip-coated samples, measured with a four-point probe system, was decreased from 800 to 65 kΩ/sq at 4 and 20 BLs, respectively (Figure 4a). The very high sheet resistance (>1 MΩ/sq) of the PANi/PEDOT:PSS thin films made by both dipping and spraying method prevented their measurement in electrical properties. The spray-assisted samples also showed dramatic decrease in sheet resistance form 80 kΩ/sq at 4 BLs to 7.8 kΩ/sq at 20 BLs (Figure 4b). Electrical conductivity was obtained by multiplying the inverse of sheet resistance by film thickness. As the number of layers deposited increased, the conductivity of both systems was gradually increased. With more cycles (or bilayers) deposited, more SWNT are effectively utilized, bridging a continuous conjugative structure. This implies that a more continuous polymer-carbon nanotubes conductive network pathway is formed with increasing thickness, providing more efficient electron transport.

Compared to the traditional dip coating method, the electrical conductivity of PANi/SWNT-PEDOT:PSS thin films prepared by spraying approach was more greatly enhanced. The maximum conductivity of 14.3 S/cm was achieved at 20 BLs, which is 8 times larger than the samples assembled by the conventional LbL dipping method (1.8 S/cm). These results indicate that the spraying method creates a more efficient electron conduction network during deposition. Polymer-based composites, composed of graphene or carbon nanotubes, become electrically conductive when their concentration is above the percolation threshold [46]. In the sprayed PANi/SWNT-PEDOT:PSS thin films, the electrical conductivity leveled off to 14.1 S/cm at 24 BLs. This suggests that the present system has a SWNT concentration above the percolation threshold with a uniform alignment of the three-dimensional network structure.

**Figure 5:**
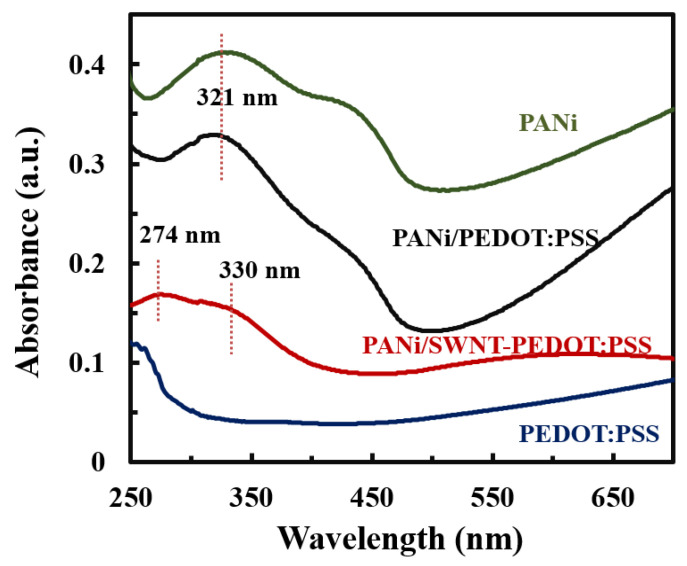
UV–vis spectra of aqueous PANi, PEDOT:PSS, PANi/PEDOT:PSS, and PANi/SWNT-PEDOT:PSS thin films.

A highly conjugated carbon network was confirmed by UV-vis spectroscopy. Figure 5 shows the UV-vis spectra of aqueous solutions of PANi and PEDOT:PSS and sprayed thin films with and without SWNT deposited. The two absorption peaks at 274 and 330 nm were observed in the UV-vis spectra. The first absorption band is assigned to the aromatic rings from SWNT and PEDOT:PSS [10,12]. A peak at 321 nm corresponds to the π–π* transitions in benzenoid units of the conducting emeraldine state of PANi [47]. This absorbance peak was red-shifted to 330 nm wavelength in the sprayed PANi/SWNT-PEDOT:PSS thin films, which indicates expanded conjugation length due to the strong π–π interfacial interaction of PANi oriented along the SWNT [48].

**Figure 6:**
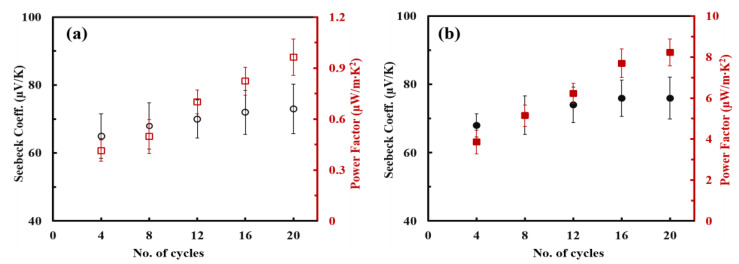
Seebeck coefficient and power factor of PANi/SWNT-PEDOT:PSS thin films that are coated by (**a**) dipping and (**b**) spraying methods.

The Seebeck coefficient as a number of deposition cycles was investigated, as shown in Figure 6. Instead of calculating *ZT* value, the power factor, in the form of *S^2^·*σ, was used to evaluate the TE performances because it is hard to measure the accurate thermal conductivity of the thin films less than 1 µm [49,50]. The values of Seebeck coefficient are positive, indicating a p-type TE material with hole-dominated carrier transport. The nanocomposites via both deposition methods exhibited a slight increase in Seebeck coefficient with layers added (dip coating: 65 µV/K at 4 BLs to 72 µV/K at 20 BLs; spray coating: 68 µV/K at 4 BLs to 76 µV/K at 20 BLs). Intrinsically, semiconducting nanotubes are n-type, but they are converted to p-type in the air because they are highly susceptible to oxygen doping [51]. Therefore, the Seebeck coefficient of n-type organic semiconductors, including carbon nanotubes (CNTs), becomes positive over time due to the electron withdrawal effects by the absorbed oxygen molecules on nanotube [52,53]. In the present study, the PANi/SWNT-PEDOT:PSS nanocomposites made from dipping and spraying methods exhibited a p-type behavior. Their thermoelectric properties, such as the electrical conductivity and Seebeck coefficient, remained stable over time under ambient conditions. Based on the electrical conductivity and Seebeck coefficient, the power factor of PANi/SWNT-PEDOT:PSS thin films was calculated as a function of layers deposited. In a similar manner to electrical conductivity, the power factor of both systems increased with increasing layers. The spray-assisted LbL films had a power factor of 8.2 µW/m*·*K^2^, which is almost 9 times as large as the same films made by the traditional LbL dip coating (0.93 µW/m*·*K^2^).

The focus of the present study is to introduce the spray-based coating concept and its baseline TE performance with comparison to the traditional LbL-dip method. Fewer layers in spray-LbL showed similar properties (growth behavior and electrical properties) in a faster deposition rate. Although the TE properties of the present study is relatively lower as compared to the previous studies that are fabricated via the spraying process, we believe that either post-treatment with acids such as concentrated H_2_SO_4_ or secondary doping could increase the electrical conductivity and, hence, the power factor [54,55,56,57]. In the present study, the PANi is only partially doped, and much higher conductivities could be realized in the LbL process by further doping in low-pH solutions of HCl or methane sulfonic acid. In this work, we used a pH 2.5 PANi solution, and it was not further doped by HCl or methane sulfonic acid. There are some reports regarding the additional improvement of electric conductivity in PANi such as the use of camphor sulfonic acid (CSA) [58]. Additionally, different molecular packing states of PANi (from compacted coil to expanded coil) can be controlled by the dissolution of the CSA-doped PANi into m-cresol by tuning the m-cresol content in the solvent [59]. The influence of further doping in low-pH solutions of HCl or CSA and of the preparation of PANi mixed with m-cresol on the thermoelectric properties is now being studied. We expect that both the doping step and the solvent effect will reveal the intrinsic correlation between the thermoelectric properties and the molecular chain structure.

### 3.3. Multilayer Structure

**Figure 7:**
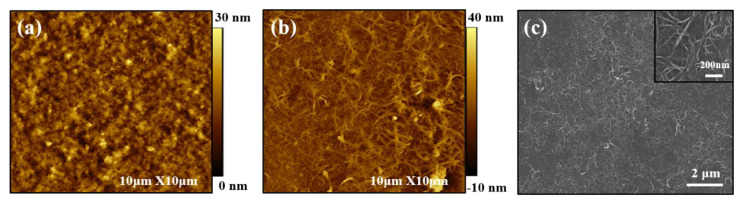
AFM height images of (**a**) PANi/PEDOT:PSS and (**b**) PANi/SWNT-PEDOT:PSS, and (**c**) SEM micrograph of 20 BL PANi/SWNT-PEDOT:PSS thin films deposited on glass slides via spray-ing method. The inset in the SEM image shows higher resolution of the SWNT network.

In order to confirm the surface morphology of fabricated nanocomposite thin films, atomic force microscope (AFM), and scanning electron microscope (SEM) analyses were carried out. Figure 7a,b represent AFM images of PANi/PEDOT:PSS and PANi/SWNT-PEDOT:PSS thin films via the spray coating method, respectively. The sprayed-PANi/PEDOT:PSS thin films exhibited a continuous and featureless surface structure. However, in the case of the SWNT-incorporated thin film, the AFM-generated surface structure of PANi/SWNT-PEDOT:PSS assembly revealed what appear to be individual carbon nanotubes and their bundles. Continuously interconnected polymeric SWNT networks were formed on the surface. The SEM image of the spray-coated multilayer thin films showed a network structure consisting of intertwined carbon nanotubes (Figure 7c). These surface structures further highlight the feasibility of the spray coating approach to obtain functional thin films without sacrificing highly dispersed carbon nanotube networks.

### 3.4. Thermoelectric Behavior

**Figure 8:**
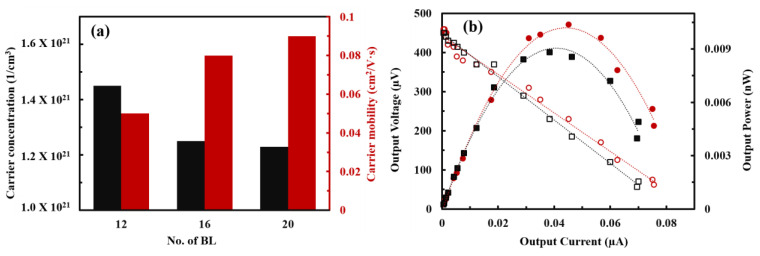
(**a**) Carrier concentration and carrier mobility of PANi/SWNT-PEDOT:PSS thin films assembled via spraying method as a function of bilayers deposited. (**b**) Output voltage (open symbols) and output power (closed symbols) as a function of current for 16 (squares) and 20 (circles) BLs PANi/SWNT-PEDOT:PSS prepared with spraying method.

Interestingly, the electrical conductivity and Seebeck coefficient in the PANi/SWNT-PEDOT:PSS thin films assembled by the spraying method were decoupled, which is an unusual observation in conventional inorganic-based TE materials. To reveal such a rare observation of a simultaneous increase in both electrical conductivity and Seebeck coefficient, carrier concentration and carrier mobility were analyzed by using Hall effect measurements in van der Pauw geometry at room temperature (Figure 8a). By increasing the number of deposition cycles, the carrier concentration of the sprayed-multilayers was decreased from 1.45 × 10^21^ cm^−3^ at 12 BLs to 1.23 × 10^21^ cm^−3^ at 20 BLs. However, the carrier mobility was increased with the number of deposition cycles (0.05 cm^2^/Vs at 12 BLs to 0.09 cm^2^/Vs at 20 BLs). The reason for a simultaneous increase in TE properties is thought to be mainly driven by enhanced carrier mobility. This is consistent with the results observed where the polymer nanocomposites, compounded with carbonaceous materials, have numerous interfaces that impede low energy carriers and facilitate the transport of the high energy counterparts [10,12]. Therefore, an energy filtering effect formed in the highly ordered structure of the multilayer assembly with single-digit nanometer-thick layers improves the power factor.

In an effort to see the applicability of organic TE materials to the industrial utilization, we have sprayed each component onto the PET substrates and measured the output voltage and power of the coated multilayer assemblies. Figure 8b shows the output performance (i.e., output voltage, output current, and output power) in the spray-coated PANi/SWNT-PEDOT:PSS thin films. When incident resistance and internal resistance values were corresponded, the maximum power factor of nanocomposites can be observed [60]. The output voltage and power of spray-coated multilayers were increased with the layers deposited. The maximum power output was 0.01 nW at a temperature gradient of 6.1 K in the 20 BLs PANi/SWNT-PEDOT:PSS thin films. Although this performance is very low for the application of organic TE, the power output can be maximized by assembling multi-module devices with a sufficiently large temperature gradient.

## 4. Conclusions

The spraying LbL deposition method was employed to fabricate the multilayer thin films by alternately depositing PANi and SWNT, stabilized in aqueous PEDOT:PSS solutions. The spray-assisted PANi/SWNT-PEDOT:PSS systems showed greater growth rate with much shorter deposition times relative to the same ones constructed by traditional dip coating. A highly interconnected and layered SWNT architecture was observed, which is commonly associated with the classical immersion process. A 20-bilayer PANi/SWNT-PEDOT:PSS thin film yielded a power factor of 8.2 μW/m*·*K^2^, which is 9 times as large as the same films made with a conventional dip coating method. The experimental parameters including spraying times, spraying distance on the receiving surface, and concentration of each component are crucial conditions to optimize the present work in terms of TE performance. A manuscript is currently in preparation for evaluating these spraying conditions for both physical properties and impact on TE behavior. This spray-based LbL assembly is expected to be applicable for large-scale industrial use due to its simple operation, versatility, and rapid processing that produces multifunctional coatings without sacrificing the advantages of the conventional dipping method.

## Data Availability

The data presented in this study are available on request from the corresponding author.
